# Trends in visits to a 24-hour walk-in crisis mental health centre during the COVID-19 pandemic

**DOI:** 10.3389/frhs.2025.1416164

**Published:** 2025-07-02

**Authors:** Jocelyne Lemoine, Depeng Jiang, Tanvi Vakil, James M. Bolton, Jennifer M. Hensel

**Affiliations:** ^1^ Department of Psychiatry, Rady Faculty of Health Sciences, University of Manitoba, Winnipeg, MB, Canada; ^2^ Department of Community Health Sciences, Rady Faculty of Health Sciences, University of Manitoba, Winnipeg, MB, Canada; ^3^ Max Rady College of Medicine, Rady Faculty of Health Sciences, University of Manitoba, Winnipeg, MB, Canada

**Keywords:** COVID-19, coronavirus, emergency psychiatry, community mental health services, virtual care

## Abstract

**Introduction:**

The sudden onset of the COVID-19 pandemic in the spring of 2020 introduced new stressors and exacerbated existing ones, which for many negatively impacted mental health or aggravated prior mental illness. As such, access to crisis care services was necessary and potentially increased, alongside public fears about virus contagion and stay-at-home public health orders. In Manitoba, Canada, visit rates were examined at a 24-hour mental health Crisis Response Centre (CRC) that offered in-person and virtual crisis assessments in a stepped care model during the COVID-19 pandemic.

**Methods:**

All visits from the three years prior to the pandemic until September 28, 2022 were retrieved from the electronic patient record. Mean weekly visits had the pandemic not occurred were predicted with an autoregressive integrated moving average model and compared with observed rates.

**Results:**

Total pre-pandemic CRC visits (14,280) decreased 22.1%–11,122 total post-pandemic CRC visits. Visit rates remained lower than predicted throughout the observation period, with the total number of visits reduced by an average of 34.1 per week (*p* < .001) during the first pandemic wave, and that gap narrowing to an average of 18.9 visits per week (*p* = 0.001) during the fourth wave. Thirteen percent of pandemic visits were virtual; highest during the first wave (average of 34.1% of visits per week) and decreased to an average of 5.6% of visits per week during the last measured period.

**Discussion:**

Further investigation is necessary to better understand this sustained pattern of reduced service utilization as we move beyond the pandemic.

## 1 Introduction

There is no debate that the COVID-19 pandemic has had an impact on the mental wellness of the public as a result of stress related to quarantine, health anxiety, and financial hardship (1, 2). Both survey and observational cohort studies from early in the pandemic reported increased symptoms of anger, anxiety and depression along with incident diagnoses (1, 3, 4). Subsequent systematic reviews reported elevated levels of depression and anxiety, especially among individuals who are female, were quarantined, experienced chronic illnesses, or infection with the COVID-19 virus (5–8). Youth seemed to be significantly affected, with elevated levels of depression and anxiety, as well as psychological distress, negative affect, and loneliness (9). Likewise, reviews examining the mental health of healthcare workers during that period reported greater incidence of and more severe symptoms of depression and anxiety, notably for female and frontline healthcare workers (10, 11). Additionally, health systems had to rapidly adapt in order to ensure continuity of care (12). Care avoidance, and some gaps in care availability, led to reduced rates of service use for mental health during the early part of the pandemic (13, 14). While outpatient services were able to rapidly transition to virtual-based services (15), the acuity and lack of guidelines for the use of virtual care for emergency and crisis intervention required the majority of these services to continue in person (16).

The literature on trends in mental health crisis care and emergency department (ED) service use during the pandemic continues to grow. Many early studies used prior year data to examine changes in visit rates, showing that, compared to the previous year, mental health presentations significantly decreased during the early part of the pandemic when public health initially ‘locked down’ and encouraged stay-at-home orders (17–19). Few studies have examined longitudinal trends in visits to mental health-specific crisis settings. Those that have used time series analyses of visit rates at dedicated mental health treatment facilities have also demonstrated reductions in visits consistent with the pre-post studies (20–23).

Studies have continued to follow visit rates further out from the pandemic onset, with Sweeny et al. investigating ED visits at 105 general hospitals in Australia from 2018 until June 2021. In the first 4 months of the pandemic, visits were 27.3% lower than expected and did not recover to predicted levels by study end despite relaxed pandemic restrictions. Similarly, a study in France (22) at a dedicated psychiatric hospital offering both in person and virtual care found a 20% decrease in emergency visits in 2020 compared to 2019, followed by a general increase in visits, which returned to, but did not surpass pre-pandemic numbers by the end of 2021 (22). Studies extending into the second half of the pandemic are still emerging to determine if use of crisis services has started to shift in keeping with the levels of persisting societal negative mental health being reported in survey studies (1, 3).

The purpose of this study was to examine longitudinal service use trends at a 24-hour walk-in mental health crisis centre in Winnipeg, Canada that remained fully operational during the COVID-19 pandemic. In addition, the centre offered virtual mental health and psychiatric assessments to provide additional access for those not wanting to attend in person for any reason (24). In this study, a longer follow-up period is offered, examining visit rates to the end of September 2022. The authors hypothesized that visits might decline initially, then return to expected levels, or even surpass usual rates as the pandemic progressed.

## 2 Methods

n this retrospective, observational study, visits to the Crisis Response Centre (CRC) in Winnipeg, Manitoba, Canada were examined between March 19, 2020 (start of first local wave and corresponding lockdown) to September 28, 2022. The CRC functions as an alternative to the general hospital emergency department for adults (age 18 and over) requiring urgent mental health assessment (MHA). It is a voluntary facility that serves the entire city of Winnipeg (population∼700,000), offering 24/7 MHA by allied health professionals and psychiatric services in a stepped care model. It also operates a 24-hour mobile crisis line. The CRC is a provincially funded institution and all services are provided free-of-charge to anyone attending, regardless of citizenship or insurance status. The population accessing services is very sociodemographically diverse. Individuals must be presenting for service voluntarily, but can self-present or attend accompanied by supports or law enforcement. Individuals may present in any kind of mental health crisis with a large range in acuity of presentations, most commonly mood and anxiety related, with an important percentage of presentations featuring personality disorders and psychosis (25). The CRC is located adjacent to a general hospital so if an individual is felt to be at immediate risk of harm to themselves or others, or presents as medically unstable, they are likely to be directed to attend more secure and medically managed facilities. Approximately, 20%–25% of individuals presenting to the CRC will require a psychiatric assessment with about half of those being admitted to hospital (25). If an individual requires hospitalization, that service is separately covered by provincial health plans or self-pay for non-insured. The CRC remained fully operational during the pandemic; clients could self-refer by walking in, attend with police services, or be referred following a call to the crisis line. Virtual MHAs and/or psychiatric consultation were offered to those who were triaged as lower acuity on-site or called the crisis line and could safely remain in the community for the assessment to occur (24).

All CRC visits where an MHA and/or a psychiatric assessment (either virtually or in person) occurred during the observation period and the three years prior from the electronic patient record were retrieved. The CRC utilizes an independent electronic patient record that includes information on every single visit that occurs. When an individual registers for service, be it in person or via contact with the mobile crisis telephone line, a visit is opened and timestamped for that individual in the electronic patient record. Any visits opened in error or miscoded are identified during monthly data audits and are corrected or deleted from the system. Based on these procedures, the electronic patient record data are considered very complete and accurate with respect to visit occurrences which are the data points utilized in the study. For the analysis, key pandemic periods were designated to correspond to the local waves of increased viral transmission and heightened public health restrictions (“Waves”). Visit rates were examined during and in-between these periods. The total and mean number of visits per week in each pandemic period were calculated. Weekly visit counts from three years before the pandemic were imported into SAS 9.4 and used for the time-series analysis. An autoregressive integrated moving average [ARIMA(1,0,1)] model was identified as the best model to describe the trends in total visits proceeding the pandemic. This ARIMA model was estimated again over the entire time period to predict the average number of visits per week in each pandemic period had the COVID-19 pandemic not occurred, and to examine the impact of the COVID-19 waves. The observed visit rates were compared with these predictions.

The University of Manitoba Health Research Ethics Board approved the study [HS23878(H2020:196)]. Informed consent from patients was not required as the research was a secondary analysis of de-identified and aggregated visit counts.

## 3 Results

**Figure 1:**
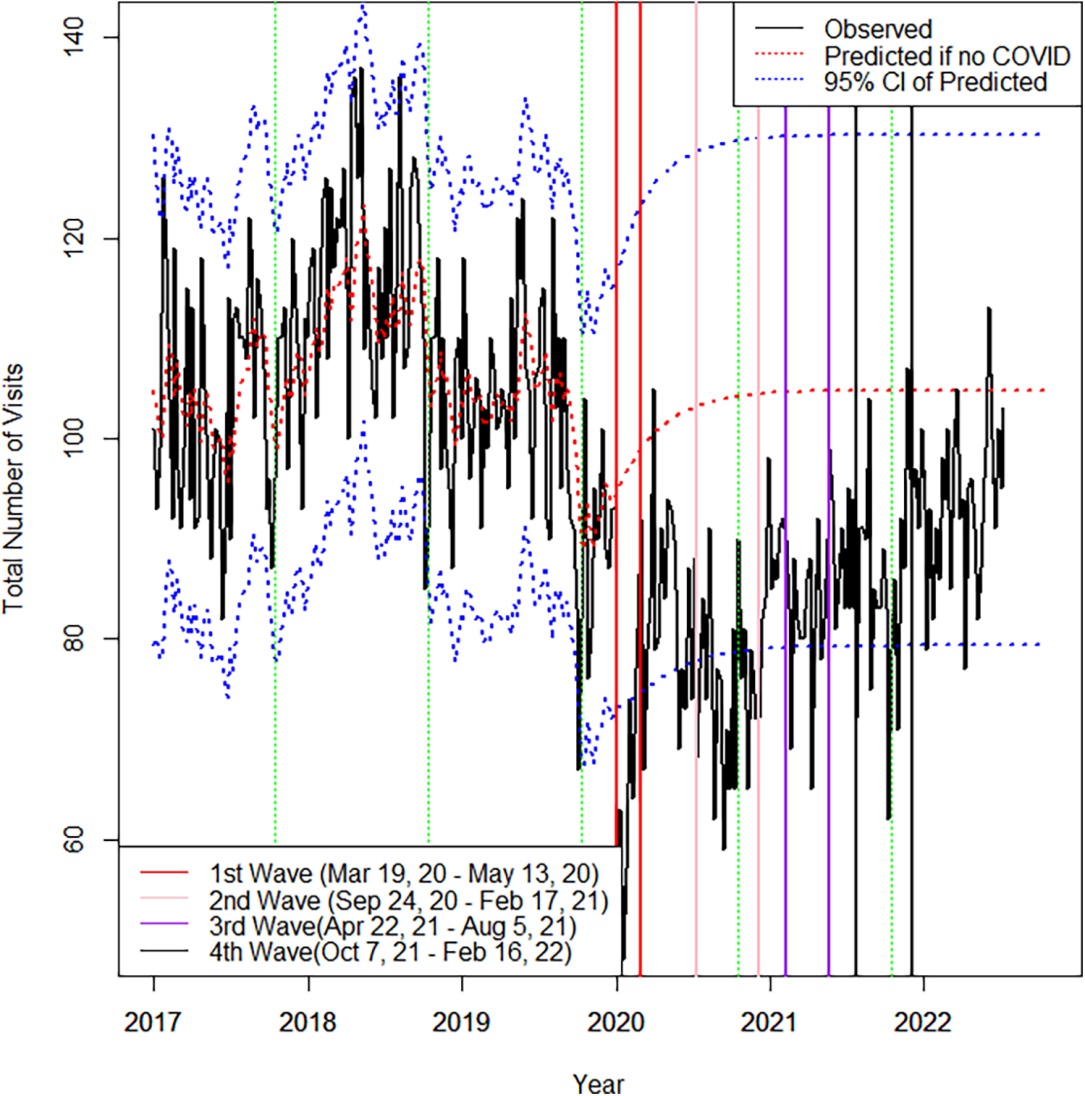
Observed and predicted total visits by week. Used/modified with permission1. CC BY 4.0 License (28). Note: Green vertical dashed lines represent the last week of each calendar year. Dotted redline represents the predicted the total number of visits if no COVID.

There were 11,122 visits in the 30 months of the pandemic examined, compared to 14,280 visits in the equivalent time period over 2017–2019, reflecting an absolute decrease of 22.1%. During the 3 years pre-pandemic the weekly visit rate was 105.9 (*SD* = 12.8). Compared to predicted rates based on the ARIMA model for the 3 prior years, weekly visit rates were lowest in Wave 1 (Figure 1), with 65.1 (*SD* = 11.4) visits per week, down an average of 34.1 (*p* < .001) from predicted. Weekly visit rates subsequently increased but remained significantly lower than predicted through the period between Wave 1 and 2 at 21.6 visits lower than expected (*p* < .001), Wave 2 at 28.5 visits lower than expected (*p* = .003), between Wave 2 and 3 at 18.3 visits lower than expected (*p* = .006), Wave 3 at 21.2 visits lower than expected (*p* < .001), between Wave 3 and 4 at 17.6 visits lower than expected (*p* = .009), and Wave 4 at 18.9 visits lower than expected (*p* = .001). After the fourth Wave, from February to September 2022, total visits were increasing but still significantly lower than expected by 12.7 visits per week on average (*p* = .02). In total, 12.8% (*n* = 1,426) of visits during the pandemic occurred virtually with the remainder in-person. Virtual visits were highest at the start of the pandemic during the first Wave accounting for an average of 34.1% (*SD* = 5.6) of weekly visits. Virtual visits subsequently decreased to an average of 5.6% (*SD* = 2.7) of weekly visits during the period after the fourth Wave until study end.

## 4 Discussion

Similar to what was observed in other centres, the substantial reduction in visits during the first COVID-19 wave mirrors the introduction of public health restrictions and peak public anxiety about the virus (17, 19, 21, 22). However, contrary to our hypothesis that visits would rebound and possibly surpass usual rates due to the rising psychological impact of the pandemic, CRC visits increased but remained lower throughout the 30 months examined. This is despite the CRC being a mental-health specific facility with the novel offering of virtual visits as a strategy to address care avoidance and provide person-centered options for crisis mental health assessment. This type of sustained decrease was also observed at dedicated mental health emergency settings in Sweden (20) and France (22) over 10 and 22 months of pandemic, respectively.

It is challenging to reconcile how the population burden of negative mental health due to the pandemic was perceived to be increasing among Canadians (26), but visits to our crisis centre were still below historical trends. Certainly, early on in the pandemic, crises could have been preferentially self-managed on the balance of fear about attending at the healthcare facility and direction from authorities to limit attendance in public settings (14). Indeed, individuals’ assessment of the risk related to seeking in-person care in primary care settings during the COVID-19 pandemic was influenced by anxiety, fear, uncertainty, and lack of information, which consequently affected individuals’ decision-making around accessing healthcare in-person (27). A higher number of individuals may have opted to call our crisis line to receive brief support as opposed to attending for a comprehensive MHA. Studies elsewhere have observed an increase in crisis calls in parallel with reduced in person service use (17, 18). What was unique about our program was the rapid adoption of virtual MHAs so that if someone needed a more comprehensive assessment and didn't want to attend in person, this could still occur and these were included in our visit counts. Other work by our team has examined characteristics of those individuals who opted for this type of assessment noting overall lower acuity indicators and time of day factors (25). As time went on and restrictions lessened, visits would have been expected to increase back to pre-pandemic levels or even higher. The CRC is the only dedicated mental health facility of its kind in the city, and has the highest volume of mental health visits across the city, so even a proportion of individuals seeking care elsewhere is unlikely to account for the sustained decreases seen. It appears, that despite need, care avoidance may have continued (14). At the conclusion of this study, the pandemic had not yet been declared over and COVID-19 precautions and screening protocols were still mandatory in our facility. This means that additional rises in visit rates with longer follow-up periods may still be seen.

Additionally, an important change that occurred during the pandemic is the emergence of virtual options making primary care and other mental health services more accessible, particularly during daytime hours, possibly diverting after hours crisis visits. New options for self-management and treatment have also emerged. For example, several months into the pandemic the Government of Manitoba temporally invested in some virtual therapy options including a cognitive behavioral therapy program and virtual counselling sessions that were made available free to all Manitobans. Some individuals who would otherwise present seeking referrals to services for lower acuity needs could have found these resources sufficient. Moreover, many organizations have launched virtual programs during the pandemic offering more options for individuals seeking help. Additionally, of note, in late 2021 our region launched an Alternative Response to Citizens in Crisis service that paired police with mental health clinicians focussed on achieving crisis resolution in community.

Limitations in this study include the lack of data on crisis calls that could have functioned as sufficient in certain situations and averted the need for a comprehensive MHA that otherwise would have occurred pre-pandemic. We also lacked data on presentation severity and how that might have changed with the pandemic. Data are also limited to the CRC and cannot be compared to what was happening with mental health crisis visits at the system level, such as visits to the surrounding general hospital EDs. Visits across specific demographic or diagnostic groups were not examined; where others have noted escalating patterns of care use among some groups including young females (22) and substance use disorders (20). Our analysis is subject to several important limitations related to the modeling approach. The ARIMA(1,0,1) model assumes that historical patterns would have persisted in the absence of the pandemic and that it adequately captures the underlying structure of the data. This may not account for unforeseen external shocks or changes in healthcare utilization patterns unrelated to COVID-19. Although we conducted diagnostic checks and selected the best-fitting ARIMA model, alternative model choices or inclusion of covariates might have altered the predicted trends. Furthermore, while we have provided 95% confidence intervals of prediction to better reflect the uncertainty of our estimates, there may still be unmeasured sources of variability that are not captured in these intervals. Thus, caution is warranted when interpreting the magnitude of the pandemic's estimated impact.

## 5 Conclusions

IEither or both the need for crisis care and care-seeking behavior changed markedly during this pandemic. More about the implications of these findings, and the identification of unmet need as a result, requires a more nuanced understanding. The role of various other contextual factors–use of community-based services, availability of virtual primary care and counselling appointments, alternative Government-funded and virtual self-directed or self-referral options made available, and the possibility of persistent care avoidance–contribute. Answering these questions and evaluating the longer-term impact of the pandemic on the mental health of the population will continue to be a priority for psychiatric services research to inform the optimal organization of mental health care.

## Data Availability

The data analyzed in this study is subject to the following licenses/restrictions: the datasets generated and/or analyzed during the current study are available from the corresponding author on reasonable request. Requests to access these datasets should be directed to Jennifer Hensel, jhensel@hsc.mb.ca.
